# Sweet taste liking is associated with impulsive behaviors in humans

**DOI:** 10.3389/fnbeh.2014.00228

**Published:** 2014-06-17

**Authors:** Jessica Weafer, Anne Burkhardt, Harriet de Wit

**Affiliations:** Department of Psychiatry and Behavioral Neuroscience, University of ChicagoChicago, IL, USA

**Keywords:** sweet taste, reward sensitivity, impulsive choice, impulsive action, delay discounting, decision-making, sucrose

## Abstract

Evidence from both human and animal studies suggests that sensitivity to rewarding stimuli is positively associated with impulsive behaviors, including both impulsive decision making and inhibitory control. The current study examined associations between the hedonic value of a sweet taste and two forms of impulsivity (impulsive choice and impulsive action) in healthy young adults (*N* = 100). Participants completed a sweet taste test in which they rated their liking of various sweetness concentrations. Subjects also completed measures of impulsive choice (delay discounting), and impulsive action (go/no-go task). Subjects who discounted more steeply (i.e., greater impulsive choice) liked the high sweetness concentration solutions more. By contrast, sweet liking was not related to impulsive action. These findings indicate that impulsive choice may be associated with heightened sensitivity to the hedonic value of a rewarding stimulus, and that these constructs might share common underlying neurobiological mechanisms.

## Introduction

There is increasing evidence that the apparently disparate psychological constructs of reward sensitivity and impulsivity have common biological origins (Jentsch and Pennington, 2014). Sensitivity to rewarding stimuli (e.g., drugs of abuse or sucrose) is typically assessed in animals by self-administration and in humans by subjective reports of liking. Impulsivity is assessed in both animals and humans using behavioral tasks of delay discounting (impulsive choice) and behavior inhibition (impulsive action). In studies with both humans and nonhuman species, greater hedonic value of rewarding stimuli has been associated with greater impulsive behavior, including both impulsive choice and impulsive action (e.g., Perry et al., 2007; Diergaarde et al., 2008, 2009; Weafer and De Wit, 2013). These associations are important because they may help to uncover the psychological and biological processes underlying risk for pathological behaviors such as substance dependence.

Preclinical evidence suggests that sensitivity to rewarding stimuli is associated with impulsive choice, as measured by discounting of delayed rewards. Perry et al. (2007) examined delay discounting for food and cocaine reward in rats bred for high or low preference for saccharin. Rats that preferred saccharin more strongly also discounted delayed food reward more steeply, but no association was found between saccharin preference and discounting of a cocaine reward. In other studies, discounting was assessed as a predictor of drug self-administration, and steeper discounting was associated with greater self-administration of alcohol, nicotine, and cocaine (Poulos et al., 1995; Perry et al., 2005; Diergaarde et al., 2008; Anker et al., 2009). The greater self-administration suggested that the more impulsive animals valued rewards more (although it is important to note that additional factors likely contribute to drug self-administration; Stephens et al., 2010).

Preclinical studies also indicate that sensitivity to reward is associated with impulsive action, or behavioral inhibition. Impulsive action is commonly measured by inhibitory errors on a go/no-go task (i.e., response inhibition) or premature responses on the 5-choice serial reaction time task (5-CSRTT; waiting inhibition). In one study, rats selected for high saccharin preference exhibited greater impulsive action on a go/no-go task when cocaine was used as the reinforcer (although not with food as the reinforcer) (Anker et al., 2008). In another study (Diergaarde et al., 2009), rats high in impulsive action on the 5-CSRTT consumed more sucrose in a self-administration task, and responded more to sucrose-associated cues than low impulsive action animals. In parallel studies with drug reward, rats high in impulsive action self-administered cocaine and nicotine at higher rates than did rats low in impulsive action (Dalley et al., 2007; Diergaarde et al., 2008; Cervantes et al., 2013), consistent with the idea that the drugs have greater reward value in these animals.

There is also indirect evidence from studies with humans that sensitivity to drug reward is associated with impulsive behaviors. For example, individuals who abused (and presumably liked) opiates, cocaine, nicotine, and alcohol discounted delayed rewards to a greater extent than did non-drug abusers (MacKillop et al., 2011; Bickel et al., 2014), although in these studies it is not clear whether the greater discounting pre-dated, or was a consequence of the drug use. Additionally, stimulant and alcohol abusers exhibited greater impulsive action than non-users (Fillmore and Rush, 2002; Bjork et al., 2004; Monterosso et al., 2005; Rubio et al., 2008), although again it is not clear if greater impulsive action in these studies was a result of drug use. Importantly, high impulsive action on the stop task was associated with greater self-reported euphoria following amphetamine (Weafer and De Wit, 2013). However, to date no studies have examined associations between hedonic response to a sweet taste and impulsive behavior in humans.

These findings suggest that individuals who experience greater reward from incentive stimuli (food or drugs) may also be more impulsive, by measures of either impulsive choice or impulsive action. In the current study, we examined associations between hedonic value of a sweet taste and two forms of impulsivity in healthy young adults. Sweet taste detection and liking were measured with a standardized task (Kampov-Polevoy et al., 1997), and the two forms of impulsivity were assessed using measures of delay discounting (impulsive choice) and behavioral inhibition (impulsive action). Based on previous findings suggesting that both impulsive choice and impulsive action are associated with sensitivity to rewarding stimuli, we hypothesized that a greater preference for sweet tastes would be associated with greater impulsive choice and greater impulsive action.

## Materials and method

### Participants

Healthy men and women (*N* = 100) aged 18–30 were recruited from the community through online and printed advertisements. Inclusion criteria included at least a high school education, fluency in English, no current or past year diagnosis on the *Diagnostic and Statistical* *Manual of Mental Disorders, Fourth Edition* (American Psychological Association, 1994), and no lifetime substance dependence (other than caffeine or nicotine). We chose to recruit healthy young adults without substance-related problems to minimize the influence of prior drug exposure on impulsive behaviors. The study was approved by the Institutional Review Board of the University of Chicago, and was carried out in accordance with the Declaration of Helsinki. Participants provided informed consent and were compensated for their time.

### Procedure

These data were obtained in the course of a larger genetic study. Participants attended a 4-h experimental session (morning or afternoon) during which they completed several behavioral tasks and self-report measures in mixed order. Participants were instructed to abstain from alcohol and drugs (other than their usual amounts of caffeine and nicotine) for 24 h before the visit, and urine samples were obtained to verify compliance. After compliance testing, participants completed the tasks reported here, which included the sweet test, delay discounting, and go/no-go task. The sweet test was conducted at least 1.5 h after the session began to ensure that participants had not eaten or brushed their teeth in the last 1.5 h.

### Measures

#### Sweet test

Subjects rated solutions of various sweetness concentrations in terms of perceived sweetness intensity and liking (Kampov-Polevoy et al., 1997, 2001). Although previous versions of the sweet test used sucrose with water, we used solutions flavored with cherry Kool-Aid. A subset of subjects (*N* = 20) was tested with both sucrose in water and sucrose Kool-Aid flavor to ensure that our procedure parallels previous reports (see Results below). Participants rated five concentrations of cherry Kool-Aid that were equivalent to the molar sucrose concentrations typically used in sweet taste liking procedures (i.e., 0.05, 0.10, 0.21, 0.42 and 0.83 M) (Kampov-Polevoy et al., 1997). The test consisted of five blocks, in which each of the five solutions were presented in random order (i.e., total of 25 taste trials). For each trial, subjects received a 2 ml serving of solution in a small opaque cup, and they were instructed to swish the solution for 5 s and then spit it out. They then rated the sweetness of the taste (from “not sweet at all” to “extremely sweet”) and their liking of the taste (from “disliked very much” to “liked very much”) on two 100-mm visual analog scales. Between trials, subjects rinsed and spit a small amount of water, and the next trial began immediately after rinsing. Sweetness and liking ratings were calculated by averaging the ratings for each solution across the five presentations. Participants were classified as sweet likers if their liking ratings were greatest for the highest concentration solution (0.83 M). All other participants were classified as sweet dislikers (Kampov-Polevoy et al., 2001, 2003).

#### Delay discounting task (DDT)

Impulsive choice was assessed using a delay discounting task (DDT) that assesses the relative value of immediate versus delayed rewards. Participants made a series of choices (90 total) between a smaller amount of money (ranging from $10–$99) delivered immediately, and a larger amount of money ($100) delivered after a delay (i.e., 1, 7, 14, 30, 60, 90, 180, or 365 days). They were told that at the end of the session a random number would be generated and if they guessed the number correctly they would receive the amount of one of their choices. Thus, subjects performed the task knowing that there was a chance they would receive one of their choices. Indifference points were calculated based on the smallest amount of money chosen over the large reward at each delay. Response consistency was calculated at each delay to ensure that participants were performing the task appropriately, and a threshold of 75% consistency was set to indicate adequate effort. The indifference points were plotted to form a discount function, and the area under the curve (AUC) of the discount function provided the dependent measure of impulsive choice (Ohmura et al., 2006; Beck and Triplett, 2009). A smaller AUC indicates a steeper discounting curve, and therefore greater impulsivity.

#### Go/no-go task

Impulsive action was assessed using a go/no-go task that measures the ability to inhibit inappropriate responses. Go (X) and no-go (K) targets were presented on the computer screen. Participants were told to respond as quickly as possible to go targets but to inhibit their response to the no-go targets. Go targets were presented on the majority of trials (*n* = 68; 85% of trials), establishing the Go response as prepotent, and making it more difficult to inhibit when the no-go targets (n-12; 15% of trials) occasionally appeared. The number of false alarms (i.e., failures to inhibit a response to a no-go target) provided the dependent measure of interest.

#### Time line follow-back (TLFB; Sobell and Sobell, 1992)

Participants completed a retrospective time line calendar of their alcohol consumption by estimating the number of standard drinks they consumed each day over the past 4 weeks. From this we calculated participants’ average number of drinks per week and number of drinking days (total number of days alcohol was consumed).

#### Alcohol use disorder identification test (AUDIT; Babor et al., 1989)

The alcohol use disorder identification test (AUDIT) is a 10-item self-report measure that assesses patterns of drinking, dependence, and alcohol-related problems. Scores range from 0 (no alcohol-related problems) to 40 (most severe alcohol-related problems), and a score of 8 or greater is typically indicative of hazardous drinking (Babor et al., 1989).

### Data analyses

We first checked to ensure that participants were able to correctly discriminate between the sweetness concentrations (Kampov-Polevoy et al., 1997, 2003). Participants who did not generate appropriate concentration-response curves were excluded from analyses. We examined the relation between sweet taste preference and task performance in two ways: associations between individual differences in sweetness liking and impulsive behaviors were tested by correlational analyses, and between-groups *t* tests were also conducted to compare sweet likers and dislikers on both impulsivity measures.

## Results

### Sample characteristics

One hundred healthy adults took part in this study (52 men and 48 women). Data from the sweet taste test were invalid for five participants (see below), resulting in a final sample of *n* = 95. Sample characteristics are presented in Table 1. Participants were light drinkers who as a group scored below the hazardous threshold on the AUDIT. The racial make-up of the sample was as follows: Asian (*n* = 4), African-American (*n* = 4), Caucasian (*n* = 82), Hispanic (*n* = 3), and Other (*n* = 2).

**Table 1:** **Sample characteristics**.

	Mean	SD
Age	23.3	3.2
Education (years)	15.4	1.6
Drinks/week	8.3	7.8
Total Drinking Days	10.3	7.3
AUDIT	6.7	3.7

### Sweet test

Four participants were unable to discriminate between the different sweetness concentrations, and one participant found all sweetness concentrations aversive (as indicated by liking ratings below 1.5 out of 100 for each). Data from these participants were excluded.

**Figure 1:**
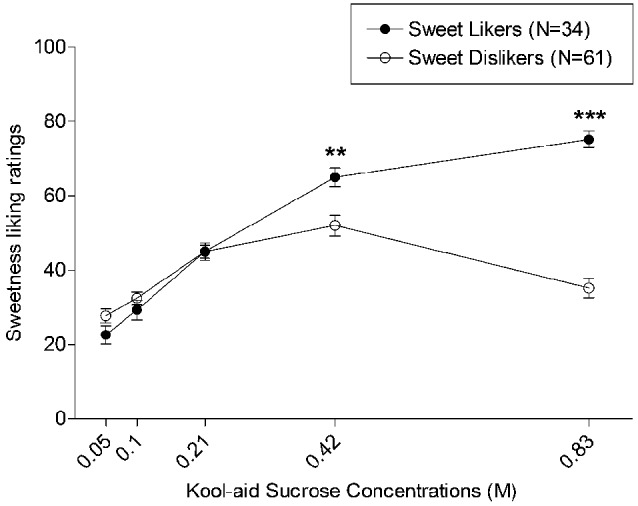
**Mean liking ratings of each of the five sweetness concentrations for sweet likers and sweet dislikers**. ** indicates *p* < 0.01 and *** indicates *p* < 0.001. Capped vertical lines represent standard error of the mean (SEM).

Participants were classified as “sweet taste likers” and “sweet taste dislikers” based on their preferred sweetness concentration. Thirty-four participants (19 men and 15 women) rated their liking of the 0.83 M concentration the highest, and thus were classified as sweet-likers. The remaining participants (30 men and 31 women) were classified as sweet dislikers. The preferred concentrations among the sweet dislikers were as follows: 0.05 N = 5; 0.10 N = 7; 0.21 N = 13; 0.42 N = 36. Figure 1 presents the mean liking ratings for each of the five concentrations separately for sweet likers and dislikers. The figure shows that the groups are comparable in terms of liking of the first three sweetness concentrations, but that the sweet likers rated their liking of the 0.41 M and 0.83 M concentrations significantly greater than the sweet dislikers (*p*s < 0.01). Sweet taste likers and dislikers did not differ in terms of age, education, or sex (*p*s > 0.21).

### Sweet liking and impulsive choice

Delay discounting data were missing from two participants. Response consistency for all participants exceeded the 75% threshold. Correlations between AUC and mean liking ratings for each of the five sweetness concentration are presented in Table 2. As greater AUC indicates less delay discounting, positive correlations represent a negative relation between discounting and sweetness liking. The table shows that AUC was positively correlated with liking of the two lowest sweetness concentrations (*p*s < 0.05), indicating that steeper discounters reported *less* liking of the two lowest concentrations solutions. By contrast, AUC was negatively associated with liking of the two highest sweetness concentrations (*p*s < 0.01), indicating that steeper discounters reported *greater* liking of the high sweetness concentration solutions.

**Table 2:** **Correlations between impulsivity measures and liking of each of the 5 sweetness concentrations**.

Impulsivity Measures	Sweetness Concentrations				
	0.05	0.10	0.21	0.42	0.83
Delay discounting AUC	0.21*	0.26*	0.03	−0.33**	−0.30**
Go/no-go false alarms	−0.13	−0.08	−0.04	−0.13	−0.08

Note: * indicates significance level of p < 0.05; ** indicates significance level of p > 0.01.

**Figure 2:**
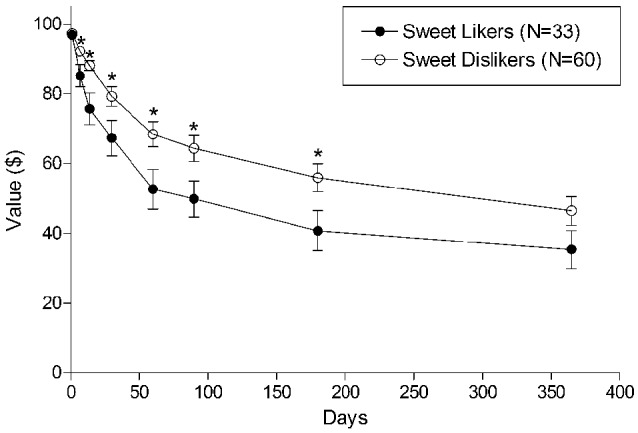
**Mean indifference points at each delay for sweet likers and sweet dislikers**. * indicates *p* < 0.05. Capped vertical lines represent standard error of the mean (SEM).

We then compared the sweet likers to sweet dislikers in regard to discounting. Indifference points at each delay are plotted for both groups in Figure 2. The figure shows that sweet likers discount delayed monetary rewards more steeply than dislikers, beginning at delays as short as one week. Mean AUC for the sweet likers was 0.46 (SD = 0.29) and mean AUC for the sweet dislikers was 0.59 (SD = 0.27). AUC was significantly smaller for sweet likers compared to sweet dislikers, *t*_(91)_ = 2.2, *p* = 0.027, indicating greater discounting of delayed rewards by the sweet likers.

### Sweet liking and impulsive action

Eight participants were missing data from the go/no-go task. Mean RT to go targets for the sample was 308.8 ms (SD = 56.6), and mean false alarms (commission errors to no-go targets) for the sample was 6.3 (SD = 3.0). Correlational analyses showed no associations between false alarms and sweet taste liking (Table 2; *p*s > 0.22). Similarly, a between-groups *t* test showed no difference between sweet likers and dislikers in false alarms on this task (*p* = 0.79).

### Associations between sweet taste liking and alcohol use

Sweet likers and dislikers did not differ on time line follow-back (TLFB) or AUDIT measures (*p*s > 0.10), and no significant correlations were observed between sweet taste liking ratings and any measures of alcohol consumption (*p*s > 0.10).

### Comparison of Kool-Aid and sucrose water solution ratings

To confirm that the Kool-Aid solutions used in the current study were comparable to the more traditional sucrose water solutions, 20 participants performed the sweet test using both types of solutions. Four participants were removed due to lack of appropriate concentration-response curves (three for Kool-Aid and one for sucrose water). Partial correlations (controlling for first solution presented; Kool-Aid or sucrose) showed significant correlations in liking ratings for the lowest concentration (0.05 M: *r* = 0.64, *p* = 0.01) and the two highest concentrations (0.41 M: *r* = 0.56, *p* = 0.029 and 0.83 M: *r* = 0.64, *p* = 0.01), suggesting that sweetness liking is comparable for both Kool-Aid and sucrose water solutions.

## Discussion

This study examined associations between preference for sweet taste and two forms of impulsive behavior: impulsive choice (delay discounting) and impulsive action (behavioral inhibition). Preference for the sweet taste was correlated with impulsive choice but not with impulsive action. Subjects who reported liking the sweetest tastes exhibited steeper discounting, and a between-groups analysis showed that sweet likers discounted delayed rewards significantly more than sweet dislikers. By contrast, no significant associations were observed between sweet liking and false alarms on the go/no-go task.

This association between delay discounting and sweet taste liking in humans parallels reports of steeper discounting in saccharin-preferring animals (Perry et al., 2007), and both animal and human studies suggesting greater sensitivity to rewarding stimuli in individuals high in impulsive choice (MacKillop et al., 2011; Carroll et al., 2013). This evidence suggests that sensitivity to reward contributes to impulsive choice. That is, greater liking of reward-related stimuli could bias decision-making in favor of immediate reinforcement, even at the expense of receiving less of the reward. Such reward-based maladaptive decision-making would likely have important implications regarding substance abuse and other pathological behaviors observed in impulsive individuals.

The lack of an association between sweet liking and impulsive action in the current study is inconsistent with some previous reports. However, most previous studies with impulsive action have been conducted with rats, using either the 5-CSRTT (Dalley et al., 2007; Diergaarde et al., 2008, 2009), or a reversal learning task (Cervantes et al., 2013). These tasks may measure different aspects of impulsive action than the go/no-go task used here. The 5-CSRTT measures a specific component of impulsive action known as waiting inhibition (i.e., the ability to wait until the appropriate time to respond), and the reversal task measures the ability to overcome previously learned responses. In contrast, the go/no-go task used in the current study measures the ability to inhibit one response in the presence of a competing response. It is possible that subtle differences in these forms of impulsive action relate differently to reward sensitivity (Jentsch et al., 2014).

The association between impulsive choice and sweet taste liking suggests that the two traits may share underlying neurobiological mechanisms. Indeed, initial findings from neuroimaging studies suggest some overlap in brain regions associated with sweet taste liking and those associated with discounting of future rewards. Administration of a sweet taste activates reward regions including the right ventral striatum and bilateral orbitofrontal cortex (Kareken et al., 2013), and sweet likers display greater activation following a sweet taste in the ventromedial prefrontal cortex (vmPFC) compared to sweet dislikers (Rudenga and Small, 2013). Similarly, both the vmPFC and ventral striatum are also implicated in processing the discounted value of future rewards (Peters and Büchel, 2011; Bartra et al., 2013), and individuals with lesions in the vmPFC display greater delay discounting (Sellitto et al., 2010). It will be important for future studies to investigate whether individuals high in impulsive choice show greater activation in these brain regions in response to both sweet tastes and other rewarding stimuli (e.g., drugs of abuse) to further understand the relation between impulsive choice and reward sensitivity.

Evidence of a genetic association between impulsive choice and sensitivity to both sweet-taste and drug-induced reward (Mitchell, 2011; Carroll et al., 2013) provides further support for common neurobiological mechanisms underlying impulsive choice and reward sensitivity. For instance, animals genetically bred to be high alcohol preferring consume greater amounts of saccharin (Sinclair et al., 1992; Stewart et al., 1994) and display steeper delay discounting than do low alcohol preferring animals (Wilhelm and Mitchell, 2008; Oberlin and Grahame, 2009). This genetic link is further supported by human studies that report an association between family history of alcoholism and both sweet taste liking and impulsive choice. That is, individuals with a family history of alcoholism are more likely to prefer sweet tastes than those without such a family history, and this is true for both alcohol dependent individuals and those with no history of drug or alcohol abuse (Kampov-Polevoy et al., 2001, 2003). Additionally, children and adults with a family history of substance abuse discount more steeply than do those without such a family history (Petry et al., 2002; Herting et al., 2010; Acheson et al., 2011).

To the extent that impulsive choice and reward sensitivity share underlying mechanisms, treatments aimed at reducing impulsive choice could potentially decrease both reward sensitivity and maladaptive reward-seeking behavior. To date, several behavioral and pharmacological methods have been identified that reduce impulsive choice. For instance, behavioral training of working memory can decrease delay discounting in stimulant addicts (Bickel et al., 2011), and reductions in impulsive choice have been observed following completion of behavioral treatment for substance abuse disorder (Landes et al., 2012). Additionally, dopaminergic drugs, including amphetamine and methylphenidate, decrease delay discounting rates in both animals and humans (Winstanley, 2011). Future studies are needed to test the degree to which such reductions in impulsive choice generalize to reductions in sensitivity to other rewards.

In sum, this study provides the first evidence of an association between impulsive choice and hedonic response to a sweet taste reward in healthy humans. Such an increased reward sensitivity is consistent with and likely contributes to the tendency of impulsive individuals to prefer the immediate delivery of smaller rewards over larger delayed rewards. This could also contribute to the increased risk of drug abuse in these individuals. Additional research is needed to investigate the neurobiological and genetic mechanisms underlying this association in order to better understand factors contributing to greater reward sensitivity in impulsive individuals.

## Author contributions

Anne Burkhardt acquired and managed the data. Jessica Weafer conducted the data analyses. Jessica Weafer and Anne Burkhardt conducted the literature review and co-wrote the first draft of the paper. Jessica Weafer, Anne Burkhardt, and Harriet de Wit designed the study and contributed to and approved the final version of the paper.

## Conflict of interest statement

The authors declare that the research was conducted in the absence of any commercial or financial relationships that could be construed as a potential conflict of interest.
